# Students’ Perspectives of the Economic, Financial, and Psychological Effects of Online Learning and Its Gender Association: A Cross-Sectional Study in Jordanian Universities

**DOI:** 10.7759/cureus.42994

**Published:** 2023-08-05

**Authors:** Odate Tadros, Shereen Arabiyat, Tamara Al-Daghastani, Nada Tabbalat, Rawand Albooz, Husam ALSalamat

**Affiliations:** 1 Department of Health Allied Sciences, Al-Balqa Applied University, Al-Salt, JOR; 2 Department of Internal Medicine, Faculty of Medicine, Al-Balqa Applied University, Al-Salt, JOR; 3 Department of Biopharmaceutics and Clinical Pharmacy, School of Pharmacy, The University of Jordan, Amman, JOR

**Keywords:** mental health, psychological effects, gender, efficiency, students' perceptions, online learning

## Abstract

Introduction: Jordan declared a quarantine in March 2020 and shut down all educational institutions in response to the COVID-19 outbreak. E-learning was a novel way to continue the educational process in the wake of the pandemic. The study evaluates online learning (OL), aiming to examine the economic, financial, and psychological effects of OL on students, as well as their concentration, university instructors' efficiency, and satisfaction with OL. This research also explores the gender association of OL effects in Jordanian universities.

Methods: This is a cross-sectional study. A self-filled questionnaire was generated using an online form and distributed to students at public and private universities through social media. The questionnaire utilized a five-point Likert scale to assess various aspects related to OL. The main evaluation involved a Chi-square test and posthoc test to examine gender-based differences in the factors associated with OL. The reliability of the questionnaire was assessed using a Kaiser-Meyer-Olkin (KMO) value of 0.721 and a Cronbach's Alpha of 0.7.

Results: A total of 720 responses were collected and analyzed. The results revealed a significant gender association with the economic/financial (P=0.001) and psychological (P=0.002) effects of OL. It was observed that 65.3% of students reported feeling depressed due to online learning. Moreover, a significant correlation was found between students' concentration and university instructors' efficiency (P=0.00). Interestingly, only 14.72% of students believed university instructors were 100% efficient in teaching theoretical and practical subjects. Additionally, 55% of the students expressed their desire for university instructors to record lectures for ease of access.

Conclusion: The study concludes that OL's poor economic/financial effects disproportionately affect female students. On the other hand, male students were more likely to strongly disagree with the poor psychological effects of OL. Moreover, female students were more inclined to strongly disagree with the notion that OL has no psychological effect. Lastly, significant findings indicate that both students and university instructors share equal responsibility for the success of OL in Jordanian universities.

## Introduction

In response to the COVID-19 pandemic, Jordan, like many other countries, faced the challenge of sustaining the educational process amidst lockdowns and social distancing measures [1,2]. As a result, e-learning emerged as a critical method to continue education after the pandemic's onset. Among the affected population, university students and academic staff at higher education institutes experienced significant stress and anxiety due to the unprecedented situation [2,3]. To address the evolving educational landscape, the Ministry of Higher Education required educational institutions to submit plans and reports outlining their policies during this period. Additionally, students were given the option to include or exclude their online grades from their cumulative GPA, reflecting the adaptability needed during this period of uncertainty.

Surveys conducted during this time highlighted that while most universities could provide "Reliable" video conferencing, issues such as fluctuating internet speeds and limited access to laptops hindered the full benefits of distance learning [4]. To meet the stringent accreditation requirements for university degrees in Jordan, educational institutions endeavored to integrate online learning with on-campus education, leading to substantial efforts to improve the quality of online teaching. Key areas of focus included enhancing the teaching/learning experience and creating digitally enhanced learning environments. Furthermore, the shift to online learning called for the adaptation of assessment methods to meet the new demands of remote education [2,5].

This study aims to evaluate online learning (OL) from the perspective of Jordanian students, focusing on various aspects of its impact. The research seeks to examine the economic, financial, and psychological effects of OL on students, along with evaluating their concentration, university instructors' efficiency, and overall satisfaction with OL. Additionally, the research delves into the gender association of OL effects in Jordanian universities. By analyzing students' satisfaction with the online learning process and its broader social and economic implications, this study aims to provide valuable insights for optimizing the efficacy and accessibility of online teaching in the context of Jordanian higher education.

## Materials and methods

Study design

A cross-sectional study design was employed to conduct a survey using a Google Form. The survey was distributed through social media platforms like WhatsApp and Facebook, reaching various segments of the Jordanian community. The study included university students from both public and private universities. The study employed a five-point Likert scale (1=strongly disagree, 2=disagree, 3=neutral, 4=agree, 5=strongly agree) to assess students' satisfaction. To ensure questionnaire validity, two clinical pharmacists and one statistician critically reviewed the drafts. Online data collection facilitated candid expressions of students' experiences with online learning, including economic barriers and practical challenges they encountered. The inclusion criteria encompassed medical students from public and private universities in all academic years. 

Sample size calculation

In this research, our estimated population consisted of approximately 500,000 students in medical fields across all academic years, attending both public and private universities. To achieve a 95% confidence level with a 5% margin of error, we conducted a sample size calculation, determining that around 400 samples were required for our survey. By obtaining 400 or more randomly selected surveys, we can confidently state that there is a 95% probability that the true value within the population falls within ±5% of the measured value obtained from the survey. To ensure the validity and reliability of our findings, we rigorously implemented random sampling techniques, which effectively minimized potential bias and allowed us to draw meaningful conclusions applicable to the broader student population in medical fields.

Ethical consideration

The Declaration of Helsinki of the World Medical Association was followed in this investigation. Al-Balqa Applied University's institutional review board (IRB) committee authorized conducting this research (Approval No. 26/3/1/176). Participants enrolled voluntarily and provided informed consent. Before commencing the questionnaire, participants received a clear explanation of the study's objectives, with a strong emphasis on privacy protection. No personal information, such as names or emails, was collected from the participants. They were directly asked whether they wished to participate in the questionnaire, and those who chose to participate were given the freedom to discontinue their involvement at any point during the process. The confidentiality and security of the study data were emphasized and ensured to be strictly accessible by the researchers.

Questionnaire items

Demographic data, including gender, age, academic year, and residence area, were collected from the participants in the local language, Arabic. The questionnaire consisted of items aimed at evaluating students' perceptions regarding university instructors' efficiency in online teaching (OT) for theoretical and practical subjects. Additionally, participants were asked to share their suggestions for improving the performance of online learning. The questionnaire also assessed various factors associated with online learning, including the financial effects of OT, its psychological impact, and its association with bullying and isolation among students. Participants were asked to rate their level of satisfaction with online learning at Jordanian universities and express their views on the pass-fail grading system during OT.

The questionnaire underwent a rigorous development process, starting with testing and refining it through multiple iterations with a focus group. After each round of revisions, the questionnaire was shared again for further testing until a semi-final version was achieved. To ensure its effectiveness, a small group assessed respondent skips, interpretations, and option comprehension. This final evaluation led to the ultimate version of the questionnaire, ready for distribution among our target population.

Data analysis

Data coding was performed using Microsoft Excel 2016 (Redmond, USA) and SPSS Inc. Released 2008. SPSS Statistics for Windows, Version 17.0. Chicago: SPSS Inc. was utilized for results evaluation. The data were analyzed at a 95% confidence interval (CI), and statistical significance was determined by p-values<0.05. Chi-square and post-hoc tests were employed for data analysis.

Content validity was ensured by subjecting each individual question in the test to expert evaluation, where specialists, including two clinical pharmacists and one statistician, assessed whether the questions accurately targeted their intended characteristics. Questions with a low content validity ratio (CVR) were subsequently excluded from the survey. To assess construct validity, statistical analyses were performed. The Kaiser-Meyer-Olkin (KMO) test was employed to determine the appropriateness of using factor analysis, while Bartlett's test was used to examine the null hypothesis of uncorrelated variables in the population correlation matrix. Components were identified based on the size of the eigenvalue, and only factors with the largest eigenvalues were considered for analysis.

## Results

Descriptive analysis

The study's sample size was 720, in which both males and females participated. Most of the students were aged 20-25. Most were from Amman, the capital city of Jordan. Also, a more significant number of students were from public universities. The demographic characteristics of the sample's age, gender, area of residence, university location and types, academic year, and educational level are shown in Table 1.

**Table 1:** Demographic data of study participants

Variables	Groups	N (%)
Gender		
	Male	178 (24.7)
	Female	542 (75.3)
Age Groups		
	17-19	280 (38.9)
	20-25	419 (58.2)
	26-30	18 (2.5)
	31-40	3 (0.4)
Residence		
	Capital City	265 (36.8)
	Middle Area	194 (26.9)
	North	145 (20.1)
	South	116 (16.1)
University Location		
	Capital City	249 (34.6)
	Middle Area	189 (19.7)
	North	142 (19.7)
	South	140 (19.4)
Specialty		
	Anesthesia and Resuscitation	3 (0.4%)
	Laboratory Analysis	13 (1.8)
	Medical Record	24 (3.3)
	Medicine	4 (0.6)
	Microbiology	2 (0.3)
	Midwife	9 (1.3)
	Nursing	84 (11.7)
	Pharmacy	581 (80.7)
University Type		
	Private University	160 (22.2)
	Public University	560 (77.8)
Study Year		
	First Year	123 (17.1)
	Second Year	541 (75.1)
	Third Year	36 (5.0)
	Fourth Year	15 (75.1)
	Fifth Year	3 (0.4)
	More Than Fifth Year	2 (0.3)
Educational Level		
	Bachelor’s Degree	162 (22.5)
	Higher Education	4 (0.6)
	Middle Diploma	554 (76.9)

Reliability statistics

Data reliability was ensured through various methods. The Kaiser-Meyer-Olkin (KMO) value and Bartlett's Sphericity test were employed to assess sample adequacy for analysis, both presented in Table 2. The obtained KMO value of 0.721 indicates superb adequacy for analysis, surpassing the recommended threshold of 0.6. Similarly, Bartlett's Test of Sphericity yielded a highly significant value of 0.000, affirming the validity and applicability of responses to the addressed problem and making the data suitable for factor analysis.

**Table 2:** The measure of sampling adequacy Minimum required Kaiser-Meyer-Olkin measure of sampling adequacy=0.5. df: degree of freedom.

Test		
Kaiser-Meyer-Olkin Measure of Sampling Adequacy		0.721
Bartlett's Test of Sphericity		
	Approx. Chi-Square	16243.063
	df	378
	p-value	0.000

Furthermore, the internal consistency of the data, evaluated using Cronbach's Alpha, was determined to be 0.66, which rounds off to 0.7. This value suggests mediocre internal consistency between the data, considering the number of factor items at 28. Overall, these reliability statistics provide confidence in the data quality and allow for robust analysis of the research findings.

Total variance explained

The eigenvalue, representing the number of extracted factors, should sum up to the number of objects factored. The analysis displays the components and their corresponding eigenvalues. The initial eigenvalues and extracted sums of squared loadings are exclusively analyzed and interpreted. For the identification of factors and selected variables, eigenvalues greater than one are considered significant.

Table 3 shows that eight components with eigenvalues greater than one were identified. Specifically, the first component has an eigenvalue of 7.931. The second component has an eigenvalue of 2.915. The third component has an eigenvalue of 1.877. The fourth component has an eigenvalue of 1.469. The fifth component has an eigenvalue of 1.334. The sixth component has an eigenvalue of 1.227. The seventh component has an eigenvalue of 1.038, and the eighth component has an eigenvalue of 1.019. Therefore, these 28 variables represent eight distinct components. Furthermore, the extracted sum of squares reveals the percentage of variance explained by each factor. The first factor accounts for 28.327% of the variance, the second factor for 10.411%, the third factor for 6.705%, the fourth factor for 5.247%, the fifth factor for 5.247%, the sixth factor for 4.765%, the seventh factor for 3.708%, and the eighth factor for 3.639%. In total, these eight components adequately represent all the characteristics or components highlighted by the 28 variables.

**Table 3:** Loading extractions of the variables required for evaluation Extraction method: principal component analysis

Component	Initial Eigenvalues			Extraction Sums of Squared Loadings		
	Total	% of Variance	Cumulative %	Total	% of Variance	Cumulative %
1	7.931	28.327	28.327	7.931	28.327	28.327
2	2.915	10.411	38.738	2.915	10.411	38.738
3	1.877	6.705	45.443	1.877	6.705	45.443
4	1.469	5.247	50.691	1.469	5.247	50.691
5	1.334	4.765	55.455	1.334	4.765	55.455
6	1.227	4.382	59.838	1.227	4.382	59.838
7	1.038	3.708	63.546	1.038	3.708	63.546
8	1.019	3.639	67.186	1.019	3.639	67.186

Factors analysis

The factor analysis in Table 4 explains each factor's variance in the communalities. For example, the variables included in the demographic section have fewer variables than others.

**Table 4:** Exploratory factor analysis Extraction method: principal component analysis. OL: online learning.

Factor Extraction	Extraction
Gender	0.398
Age	0.570
Residence	0.818
University Location	0.882
Specialty	0.291
University Type	0.340
Year	0.493
Educational Level	0.589
Positive Financial Effect Of OL	0.498
The Poor Financial Effect of OL Due to Fast Internet	0.746
The Poor Economic Effect of OL Due to Its Cost	0.744
Poor Economic/Financial Effect of OL	0.965
OL Increases Bullying Among Students	0.754
OL Increased Isolation and Depression	0.704
The Poor Psychological Effect of OL	0.963
The Positive Psychological Effect of OL	0.603
No Psychological Effect of OL	0.544
OL for Theoretical Subjects	0.676
OL for Practical Subjects	0.584
OL Success Depends on the Student's Concentration	0.548
OL Is Improved Using Audiovisual Aids	0.610
OL for Theoretical Subjects Depends on University Instructors ' Efficiency	0.716
OL for Practical Subjects Depends on University Instructors ' Efficiency	0.782
OL Depends on University Instructors ' Efficiency	0.896
OL Results Show the Actual Academic Performances of Students	0.520
OL Satisfaction in Jordan Universities	0.708
Students Satisfaction With the Pass-Fail Grading System	0.913
OL Satisfaction	0.957

Evaluation of factors affecting OL

Several associations have been assessed to clarify influences on OL during the pandemic and are summarized in Table 5. The evaluation is conducted based on the means of the estimated factors.

**Table 5:** Evaluation of potential associations for factors affecting online learning The p-value represents asymptotic two-sided significance. OL: Online learning; df: Degree of freedom; NA: Not applicable; a: 0 cells (.0%) have an expected count of less than five. The minimum expected count is 5.69; b: 1 cell (10.0%) has an expected count of less than five. Therefore, the minimum expected count is 4.70.

Comparison	Chi-Square	Value	df	P-value
Gender vs. Poor Economic/Financial Effect of OL				
	Pearson Chi-Square	18.418^a^	4	0.001
	Phi	0.160	NA	0.001
	Cramer's V	0.160	NA	0.001
Gender vs. Poor Psychological Effect of OL				
	Pearson Chi-Square	16.866a	4	0.002
	Phi	0.153	NA	0.002
	Cramer's V	0.153	NA	0.002
Gender vs. No Psychological Effect of OL				
	Pearson Chi-Square	16.596^b^	4	0.002
	Phi	0.152	NA	0.002
	Cramer's V	0.152	NA	0.002
Students' concentration vs. University Instructors' Efficiency for OL				
	Pearson Chi-Square	176.696^a^	10	0.000
	Phi	0.495	NA	0.000
	Cramer's V	0.350	NA	0.000
Gender vs. Students' Satisfaction Toward OL				
	Pearson Chi-Square	10.48	9	0.313
Gender vs. Improved Use of Audiovisual Tools for OL				
	Pearson Chi-Square	10.48	9	0.276

Association between gender and the poor economic/financial effect of OL: There is a significant association between gender and the poor economic/financial effect of OL (p-value=0.001). The strength of association, as indicated by Phi and Cramer's V, is strong (0.160). A post hoc test was conducted for further analysis, showing a significant adjusted residual (3.20) greater than the critical value (1.96) with a p-value of 0.001.

Association between gender and the poor psychological effect of OL: There is a significant association between gender and the poor psychological effect of OL (p-value=0.002). Phi and Cramer's V indicate a strong association (0.153). The post hoc test resulted in a significant adjusted residual of 3.35, exceeding the critical value (1.96), with a p-value of 0.000.

Association between gender and no psychological effect of OL: The results demonstrate a significant association between gender and no psychological effect of OL (p-value=0.002). Phi and Cramer's V reveal a strong association (0.152). The post hoc test produced significant adjusted residuals of 2.97 and 3.6, both greater than the critical value (1.96), with p-values of 0.002 and 0.000, respectively.

Association between students' concentration and university instructors' efficiency for OL: There is a significant association between students' concentration and university instructors' efficiency for OL (p-value=0.002). Phi and Cramer's V show a strong association (0.152). The post hoc test resulted in significant adjusted residuals of 4.0, 3.4, and 6.5, all exceeding the critical value (1.96) with p-values of 0.000, 0.000, and 0.00, respectively.

Association between gender and students' satisfaction in Jordanian universities for OL: The results show an insignificant association between gender and student satisfaction in Jordanian universities for OL (p-value=0.313).

Association between gender and improved use of audiovisual tools for OL: The results also indicate an insignificant association between gender and the improved use of audiovisual tools for OL (p-value=0.313).

Evaluation of students’ perceptions for university instructors ' efficiency in OT

Table 6 presents the assessment of university instructors' efficiency in teaching theoretical and practical subjects through OT. The results indicate that only a small percentage of students (14.72%) believe that university instructors are 100% efficient in delivering online lessons. Conversely, most students (56.81%) perceive university instructors' efficiency in online teaching as 0%.

**Table 6:** Evaluation of students’ perceptions regarding university instructors' efficiency in theoretical and practical subjects in online teaching, N=720. OT: online teaching; OL: online learning.

Efficiency	OT for theoretical subjects depends on university instructors' efficiency, N (%)	OT for practical subjects depends on university instructors' efficiency, N (%)	Total number of subjects believing about university instructors' efficiency for OL, N (%)
0%	107 (14.86)	302 (41.94)	409 (56.81)
25%	195 (27.08)	198 (27.5)	393 (54.58)
50%	209 (29.03)	57 (7.92)	266 (36.94)
75%	137 (19.03)	129 (17.92)	266 (36.94)
100%	72 (10)	34 (4.72)	106 (14.72)
Total	720 (100)	720 (100)	720 (100)

Evaluation of suggestions for improving performance for OL

Table 7 presents students' suggestions regarding improvements for OL. Most students expressed the desire for their university instructors to record lectures after online classes, enabling them to review the videos for better learning comprehension. On the other hand, a considerable number of students provided various suggestions for enhancing OL, but the specifics of these recommendations were not explicitly specified. Conversely, fewer students believed that reducing quizzes, assignments, or short tests was essential for improving the online learning experience.

**Table 7:** Students' suggestions for improving the performance of online learning, N=720. OL: online learning

Suggestions for Improving Performance of OL	N(%)
Record Online Lectures	399 (55.42)
Many Others	224 (31.11)
Interactive Lectures	209 (29.03)
Changing the Dates of Electronic Lectures	89 (12.36)
More Quizzes/Short Tests	66 (9.17)
Back to On-Campus	62 (8.61)
Flexible Online Lectures Time	46 (6.39)
More Homework/Assignments	41 (5.69)
Unifying the Platform	35 (4.86)
Standardization of the Platform	11 (1.53)
Platform Consolidation	12 (1.67)
Reduce Quizzes/Assignments/Short Tests	15 (2.08)

## Discussion

The present study examined the impact of OL on Jordanian university students, focusing on various factors affecting their learning experiences. Jordan boasts 40 universities, including public, private, regional, and foreign institutions, adhering to high higher education standards [6,7]. Our findings revealed a significant gender association with the economic/financial effects of OL, as many female students saved on transportation expenses but incurred additional costs for electronic devices and internet services to attend online lectures. This contradicted previous studies by Ong & Lai, which indicated that women were more influenced by perceptions of computer self-efficacy and ease of use [8], and aligned with the results of Jiang et al., who found that females were less likely than males to enroll in massive open online courses [9].

Moreover, students from disadvantaged areas encountered challenges with OL, such as poor connectivity and limited access to personal electronic devices, leading to reduced time spent studying online compared to pre-pandemic conditions [3]. As a result, 8% of students felt that the educational benefits did not justify the costs, leading them to prefer a pass-fail grading system to prevent any negative impact on their GPAs.

Loneliness, isolation, and depressive feelings were reported by students during the online learning experience, with an increase in bullying among colleagues, in line with similar findings in a Canadian study that highlighted the negative impact on students' social lives and increased need for social support services [10]. Our results also supported a significant gender association with the poor psychological effects of OL, with posthoc tests indicating that more males disagreed strongly with experiencing such effects [11]. Additionally, our study found that more females strongly disagreed with the notion of no psychological effect of OL, aligning with previous studies showing females to be more psychologically affected [12]. Magson et al. also reported significant increases in depressive symptoms and anxiety among adolescents during the transition to online learning, particularly pronounced among girls [13].

Furthermore, our evaluation of the association between students' concentration and university instructors' efficiency for OL demonstrated that both factors significantly contribute to the success of OL. These results corroborate Wenglinsky's findings, indicating that university instructors can play an equal role in student learning alongside the students themselves [14]. However, no sufficient studies were found to show a direct association between OL success dependency and student concentration. A recent study by Walters et al. indicates that pupils' learning experiences, including concentration, engagement, capacity to learn, and self-esteem from learning, were significantly lower for online learning compared to the traditional classroom setting, with the transition to online learning negatively impacting students' ability to concentrate, engage in their schoolwork, and learn, leading to decreased feelings of self-worth and mental well-being, particularly among students with specific learning difficulties [15].

Regarding student satisfaction, our study found no significant association between gender and satisfaction for OL, which contrasts with the results of Yekefallah et al. may be attributed to the difference in sample size [16]. However, our findings align with Al Azmeh's study, showing no variation in electronic services and student satisfaction based on gender, specialty, or age [17]. Suri & Sharma also reported that there was no correlation between gender and the use of different e-learning formats [18]. Nevertheless, Martin et al. found that the students were less satisfied with the learning procedure [19].

Regarding university instructors' efficiency, students reported lower effectiveness in online teaching compared to on-campus instruction for both theoretical and practical subjects. This might be attributed to the challenges and psychological distress university instructors faced during the COVID-19 pandemic [2], as supported by the study of Yasemin et al., which found that instructors' beliefs in their abilities to meet distance education requirements were not at a high level [20].

Finally, students provided suggestions to enhance online teaching, including incorporating illustrative techniques, adjusting lecture times, and recording lectures. This aligned with the findings of Nartiningrum and Nugroho, where students expressed the need for more communication and social interactions among instructors and students [21]. Similarly, Martin et al. reported low ratings for interactive visual syllabi and video-based course introductions for online courses [19]. In our study, 29% of students expressed the need for more interactive lectures, which they felt were lacking in OL. The impact of self-awareness and student engagement in online sessions on the success of OL was also recognized in previous studies [22,23].

The findings highlight the significance of recording online lectures and employing illustrative techniques as essential strategies to optimize students' perceptions and enhance the online learning process. Additionally, emphasizing the shared responsibility between students and instructors is crucial for successful online learning outcomes. To address potential economic constraints, one suggestion is to provide economical internet bundles that enable students to engage effectively in online learning. Furthermore, a combined approach of online and in-campus learning may be employed to mitigate any psychological effects associated with exclusive online learning.

Limitations

The main limitations of this study are related to the online distribution of the questionnaire, which may have resulted in unequal responses from public and private universities, limiting the representation of diverse perspectives among students in Jordan. Nevertheless, online data collection allows students to freely express their true experience regarding online learning and is the most practical way to reach out to the intended study sample. Additionally, the study's focus on students enrolled in medical-related fields may restrict the generalizability of its findings to students in other academic disciplines. To enhance the study's comprehensiveness, future research could incorporate a more balanced representation of universities and include a wider range of academic fields, enabling a more comprehensive evaluation of students' perceptions of online learning across diverse educational contexts.

## Conclusions

The current study revealed that the poor economic/financial effects of online learning predominantly impact females, whereas more males strongly disagree with the notion of poor psychological effects of OL. Furthermore, a noteworthy association was identified between students' concentration and university instructors' efficiency. The findings also indicated that only a few students believed that university instructors were 100% efficient in teaching theoretical and practical subjects during online learning. However, incorporating illustrative techniques and recording lectures emerged as potential strategies to enhance learning efficiency. Importantly, the study highlighted the shared responsibility of both students and university instructors in ensuring the success of online learning.
